# Evaluation and utility of mitochondrial ribosomal genes for molecular systematics of parasitic nematodes

**DOI:** 10.1186/s13071-020-04242-8

**Published:** 2020-07-20

**Authors:** Abigail Hui En Chan, Kittipong Chaisiri, Serge Morand, Naowarat Saralamba, Urusa Thaenkham

**Affiliations:** 1 Department of Helminthology, Faculty of Tropical Medicine, Mahidol University, Bangkok, Thailand; 2 CNRS ISEM-CIRAD ASTRE, Faculty of Veterinary Medicine, Kasetsart University, Bangkok, Thailand; 3 Department of Molecular Tropical Medicine and Genetics, Mahidol University, Bangkok, Thailand

**Keywords:** Nematodes, Molecular systematics, Phylogeny, Genetic marker, Mitochondrial ribosomal genes

## Abstract

**Background:**

Molecular advances have accelerated our understanding of nematode systematics and taxonomy. However, comparative analyzes between various genetic markers have led to discrepancies in nematode phylogenies. This study aimed to evaluate the suitability of using mitochondrial *12S* and *16S* ribosomal RNA genes for nematode molecular systematics.

**Methods:**

To study the suitability of mitochondrial *12S* and *16S* ribosomal RNA genes as genetic markers for nematode molecular systematics, we compared them with the other commonly used genetic markers, nuclear internal transcribed spacer 1 and 2 regions, nuclear *18S* and *28S* ribosomal RNA genes, and mitochondrial cytochrome *c* oxidase subunit 1 gene. After that, phylum-wide primers for mitochondrial *12S* and *16S* ribosomal RNA genes were designed, and parasitic nematodes of humans and animals from 75 taxa with 21 representative species were inferred through phylogenetic analyzes. Phylogenetic analyzes were carried out using maximum likelihood and Bayesian inference algorithms.

**Results:**

The phylogenetic relationships of nematodes based on the mitochondrial *12S* rRNA gene supported the monophyly of nematodes in clades I, IV, and V, reinforcing the potential of this gene as a genetic marker for nematode systematics. In contrast, the mitochondrial *16S* rRNA gene only supported the monophyly of clades I and V, providing evidence that the *12S* rRNA gene is more suitable for nematode molecular systematics. In this study, subclades of clade III containing various nematode families were not monophyletic when the *16S* or *12S* rRNA gene was used as the genetic marker. This is similar to the phylogenetic relationship revealed by previous studies using whole mitochondrial genomes as genetic markers.

**Conclusions:**

This study supports the use of the *12S* rRNA gene as a genetic marker for studying the molecular systematics of nematodes to understand intra-phyla relationships. Phylum-wide primers for nematodes using mitochondrial ribosomal genes were prepared, which may enhance future studies. Furthermore, sufficient genetic variation in the mitochondrial *12S* and *16S* rRNA genes between species also allowed for accurate taxonomy to species level, revealing the potential of these two genes as genetic markers for DNA barcoding.
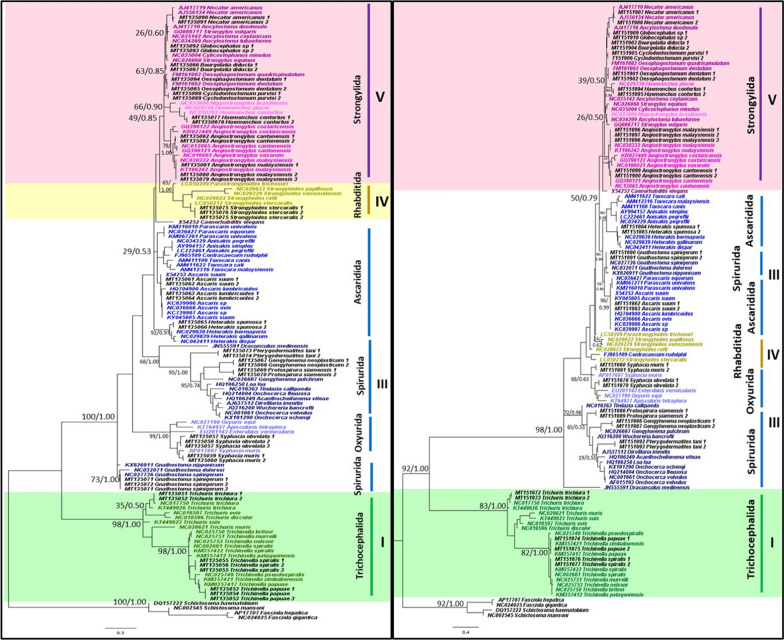

## Background

Nematodes, commonly known as roundworms, comprise the second largest phylum in Animalia, which has more than 40,000 extant species [1]. They are a highly diverse group of organisms, and of the extant species, approximately a third of them are found in vertebrates, with a significant proportion causing diseases in humans and animals worldwide [2]. Human parasitic nematodes include soil-transmitted nematodes, such as *Ascaris lumbricoides* and *Trichuris trichiura*, as well as filarial nematodes, such as *Wuchereria bancrofti* and *Onchocerca volvulus*. The World Health Organization estimates that 2000 million and 120 million people worldwide are infected with soil-transmitted nematodes and filarial nematodes, respectively [3]. Therefore, a substantial proportion of the world’s population is at risk of acquiring diseases caused by nematodes. Animal parasitic nematodes also cause substantial economic losses by infecting livestock [3].

The earliest classification of nematodes was proposed by Chitwood & Chitwood [4], in which nematodes were identified based on morphological characteristics and ecological traits. The morphological classification was based on the presence or absence of phasmids, which divided the phylum into two classes, Adenophorea and Secernentea [1, 4–6]. Subsequently, Blaxter et al. [7] used full-length nuclear *18S* rRNA sequences coupled with morphological characteristics and ecological traits for further classification within the phylum. This analysis divided Nematoda into five clades, which corroborated with the initial classification by Chitwood & Chitwood [4] and helped to provide a framework for further phylogenetic studies. More recently, mitochondrial genomes have provided an alternative picture of nematode phylogeny and increased our understanding of phylogenetic relationships among nematodes [8–10]. Park et al. [8] and Liu et al. [9] used 36 and 65 full mitochondrial genome sequences, respectively, to provide novel hypotheses for this phylum.

Comparative analyzes between phylogenies have revealed an incongruence in nematode relationships between nuclear and mitochondrial genes. The major differences occur within clade III, where the current working hypothesis, using *18S* ribosomal RNA (rRNA) gene phylogeny, indicates that it is monophyletic, whereas mitochondrial genome analysis suggests it is not monophyletic. Moreover, *18S* rRNA gene phylogenies show a close relationship between Ascaridida and Spirurida, with Oxyurida being a sister clade to the Ascaridida and Spirurida clade, whereas mitochondrial-based phylogenies show that Ascaridida is a sister clade to Rhabditida, and Oxyurida is a sister clade to the Ascaridida and Rhabditida clade [7–12]. Incongruence between phylogenies calls for the development of new genetic markers to facilitate alternative analyzes of nematode relationships.

The mitochondrial *12S* and *16S* ribosomal RNA gene sequences could be useful phylogenetic markers, as these genes have slower evolution rates compared to mitochondrial protein-coding genes [13–15]. The advantages of the mitochondrial genes include the presence of multiple copies, a lack of recombination, and higher mutation rates than nuclear genes [13, 14]. These molecular attributes potentially make mitochondrial *12S* and *16S* ribosomal RNA genes effective genetic makers for analyzing phylogenetic relationships in systematic studies of Nematoda. These genes have already been successfully used to infer and study phylogenetic relationships in snakes, frogs, crayfish, and bats [15–19]. Furthermore, mitochondrial *12S* and *16S* rRNA genes were used to study phylogenetic relationships in the nematode family Onchocercidae [20, 21]. However, phylum-wide phylogenetic analysis of nematodes has only been studied using the nuclear *18S* rRNA gene and whole mitochondrial genomes. The potential use of mitochondrial *12S* and *16S* rRNA genes as markers to analyze nematode taxonomy cannot be ignored. Therefore, in this study, we evaluated the usefulness of mitochondrial *12S* and *16S* rRNA genes for nematode systematics to provide alternative genetic markers for nematode phylogeny to gain a greater understanding of nematode relationships.

Mitochondrial *12S* and *16S* rRNA genes were evaluated in comparison with other genetic markers: nuclear internal transcribed spacer 1 and 2 regions (ITS1 and ITS2), nuclear *18S* and *28S* ribosomal RNA genes (*18S* and *28S* rRNA), and mitochondrial cytochrome *c* oxidase subunit 1 gene (*cox*1). Through these comparisons, we aimed to determine whether mitochondrial *12S* and *16S* rRNA genes are suitable for use in nematode systematics.

In this study, partial mitochondrial *12S* and *16S* rRNA sequences of human and animal parasitic nematodes from 75 taxa, including 21 representative taxa and four parasitic trematode taxa, were used to clarify nematode phylogenetic positions and provide evidence of their potential as genetic markers for nematode systematics. We also used the phylogenies of mitochondrial *12S* and *16S* rRNA genes to infer nematode relationships and increase the taxonomic breadth of parasitic nematodes. Using the mitochondrial *12S* and *16S* rRNA genes, we aimed to obtain nematode phylogenies that would allow for a more representative phylogenetic hypothesis and provide more robust support for the existing framework of nematode relationships.

## Methods

### Taxon sampling

#### Representative nematodes for mitochondrial *12S* and *16S* rRNA gene sequences

We used representative nematodes of each clade (I, III, IV and V) that had previously been collected from humans and animals in various locations of Thailand. All nematodes were preserved in 70% ethanol and stored at − 20 °C as archived specimens in the Department of Helminthology, Faculty of Tropical Medicine, Mahidol University, Bangkok, Thailand. Individual nematodes were identified, primarily to species level, based on morphological characteristics and host species using nematode taxonomic keys [2, 5, 6].

Full-length mitochondrial *12S* and *16S* rRNA gene sequences were mined from the complete mitochondrial genomes of each nematode species on the NCBI database (http://www.ncbi.nlm.nih.gov). A total of 120 sequences from 75 species were used for the analysis together with our sequence data. All DNA sequences used in this study are listed in Additional file 1: Table S1.

#### Representative nematode sequences of nuclear ribosomal ITS1 and ITS2 regions, 18S and 28S rRNA genes, and mitochondrial cox1 gene

Full length and partial sequences of nuclear ribosomal ITS1 and ITS2 regions, *18S* and *28S* rRNA genes, and the mitochondrial *cox*1 gene were mined from the NCBI database. Sequences were obtained from different genetic markers for comparison and to evaluate the suitability of mitochondrial *12S* and *16S* rRNA genes as genetic markers for molecular systematics. Each gene was evaluated using the following criteria: nucleotide substitution saturation; clade arrangement; presence of indels; and availability of full-length sequences in the NCBI database.

### Molecular analyzes

#### DNA extraction

The nematode samples were individually separated into 1.7 ml microcentrifuge tubes and washed thoroughly with sterile distilled water. For larger-sized nematodes, a small section (approximately 0.3 cm in length) of tissue was removed for DNA extraction, and a portion of the specimen was stored in 70% ethanol for further analysis. For the larval stages or smaller-sized nematodes, the whole worm was used. Total genomic DNA was isolated from each sample using the Geneaid genomic DNA mini kit (Geneaid Biotech Ltd., Taipei, Taiwan) following the manufacturer’s recommendations.

#### Primer design

Phylum-wide primers for nematode mitochondrial *12S* and *16S* rRNA genes were designed using DNA sequences obtained from NCBI. Sequences were aligned using ClustalX 2.1 [22], and conserved regions were manually checked with Bioedit 7.0 [23]. Primers were designed at the conserved region of the *12S* and *16S* rRNA genes. The properties of oligonucleotide primers, including GC content, amplicon size, melting temperature, and hairpin formation, were calculated and predicted by OligoCalc version 3.27 (http://biotools.nubic.northwestern.edu/OligoCalc.html) [24]. A gradient PCR was performed to optimize the annealing temperature for each primer set. DNA sequences and NCBI accession numbers used for primer design are provided in Additional file 2: Table S2. Clade II nematodes which consist of plant-parasitic and free-living, as well as plant-parasitic nematodes belonging to clade IV were excluded from the primer design as this study only focused on human and animal parasitic nematodes. Two different primer sets were designed: one for clade I nematodes and separate primers for clade III, IV, and V nematodes. The nucleotide sequences of primers with their respective amplicon size and thermocycling conditions are provided in Table 1.

**Table 1:** Designed primer sequences with the respective annealing temperature and amplicon size for gene amplification

Target gene	Primer name	Sequence (5ʹ-3ʹ)	Annealing temperature (°C)	Amplicon size (bp)
*12S* rRNA	12S.C1.F	GTGCCAGCTAYCGCGGTTA	55	460
	12S.C1.R	GRTGACGGGCRATATGTG		
	12S.C345.F	GTWCCAGAATAATCGGMTA	46	
	12S.C345.R	ATTGAYGGATGRTTTGTRC		
*16S* rRNA	16S.C1.F	ACGAGAAGACCCTRGRAAYT	50	240
	16S.C1.R	GRTYTAAACTCAAATCACG		
	16S.C345.F	AAGATAAGTCTTYGGAARYT	45	
	16S.C345.R	GAAYTAAACTAATATCAM G		

#### PCR and DNA sequencing

Amplification of mitochondrial *12S* and *16S* rRNA genes was performed in a thermocycler (Bio-Rad, California, USA) using the primers in Table 1. PCR was performed in a final volume of 30 µl, containing 15 µl of 2× OnePCR^TM^ master mix (GeneDireX. Inc., Taoyuan, Taiwan), 0.1 µM of each primer, and 5 ng/µl of DNA. The PCR thermocycling profiles were: 94 °C for 2 min of initial denaturation; then 35 cycles of 94 °C for 30 s, 45 °C to 55 °C for 1 min, and 72 °C for 2 min; followed by a final extension step at 72 °C for 5 min. The annealing temperatures used for each primer set are listed in Table 1. Amplicons were checked and visualized on 1% agarose gel and stained with SYBR^TM^ safe (Thermo Fisher Scientific, Waltham, USA). Successful amplicons were purified using the Geneaid PCR purification kit (Geneaid Biotech Ltd.). Sequencing for each sample was performed by a commercial company (Macrogen, Seoul, South Korea) on an automated Sanger sequencer. Mitochondrial *12S* and *16S* rRNA gene sequences generated in this study were deposited in the NCBI database (NCBI: MT135051-135093 for *12S* rRNA; MT151872-151910 for *16S* rRNA).

### Sequence alignment and phylogenetic analysis

#### Analysis of representative nematodes for mitochondrial *12S* and *16S* rRNA gene sequences

Electropherograms of the partial mitochondrial *12S* and *16S* rRNA gene sequences for all representative nematodes were manually checked using Bioedit 7.0 [23]. To generate a dataset of concatenated DNA sequences, the two partial gene sequences were manually merged. Each gene sequence was then aligned with the sequences mined from the NCBI database listed in Additional file 1: Table S1 using ClustalX 2.1 [22, 23]. The aligned sequences were manually checked and edited for ambiguous sites, and gaps were removed before phylogenetic analysis by maximum likelihood (ML) and Bayesian inference (BI). ML analysis was performed using MEGA 6.0 with the best fit nucleotide substitution model and with 1000 bootstrap replicates [25]. ML bootstrap support of > 70% was considered strong [26]. BI analysis was performed using MrBayes 3.2 [27] and was conducted using four MCMC chains for 1,000,000 generations and a sampling frequency of every 100 generations. Bayesian probability values were calculated after discarding the initial 25% of trees as ‘burn-in’. Four species belonging to phylum Trematoda were used as outgroups to root the phylogenetic trees. The phylogenetic trees were colored and edited using FigTree 1.3.1 [28], and the pairwise genetic distances (p-distance) between clades were calculated using MEGA 6.0 [25].

#### Analysis of nuclear ribosomal ITS1 and ITS2 regions, *18S* and *28S* rRNA genes, and mitochondrial *cox*1 gene sequences

We aimed to compare the robustness of the phylogenetic relationships obtained from the previously used genetic markers with the results of the mitochondrial *12S* and *16S* rRNA genes. DNA sequences of nuclear ribosomal ITS1 and ITS2 regions, *18S* rRNA and *28S* rRNA genes, and the mitochondrial *cox*1 gene that were mined from the NCBI database were aligned using ClustalX 2.1 [22, 23]. The aligned sequences were manually checked, and phylogenetic analysis of each gene was performed using the two methods mentioned above [23, 25–27]. Assessment of nucleotide substitution saturation was performed using DAMBE 6 as the criterion for genetic marker evaluation [29]. Saturation was based on the values of Iss and Iss.c, whereby Iss < Iss.c indicated that the genetic marker was not saturated and *vice versa*.

## Results

### Evaluation of potential genetic markers for molecular systematics of nematodes

DNA sequences from the NCBI database were obtained for nuclear ribosomal regions ITS1 and ITS2, *18S* and *28S* rRNA genes, and mitochondrial genes of *cox*1 and *12S* and *16S* rRNA. The DNA sequences from NCBI are provided in Additional file 1: Table S1, and a summary of each genetic marker with the respective criteria is provided in Table 2.

**Table 2:** Comparison of genetic markers with evaluation criteria and their suitability for molecular systematics of nematodes

Criteria	Nuclear gene/region				Mitochondrial gene		
	ITS1	ITS2	*18S*	*28S*	*cox*1	*12S*	*16S*
Nucleotide saturation^a^	Yes	Yes	No	No	No	No	No
Clade arrangement (monophyly)^b^	na	na	4/4	2/4	3/4	3/4	3/4
Presence of indels	High				Low		
Availability of full-length sequences	Partial sequences				Full-length sequences		

^a^Number of differences between two sequences becomes a less accurate indicator of the actual number of nucleotide substitutions. “Yes” indicates that most of the sites have already been changed before (Iss > Iss.c), indicating nucleotide saturation

^b^Number of clades (*n*) that are monophyletic out of the total number of expected monophyletic clades (e.g. n/4)

*Abbreviations*: na, not applicable

#### Nucleotide substitution saturation as an indicator of genetic marker potential

Nucleotide substitution saturation, which is an indicator of whether the genetic marker is useful, revealed that the nuclear ribosomal ITS regions were saturated. Analysis using DAMBE 6 showed Iss > Iss.c (*P* < 0.005), indicating that ITS regions are saturated and are not good genetic markers. Nuclear ribosomal *18S* and *28S* rRNA genes, as well as mitochondrial *12S*, *16S* and *cox*1 genes, were not saturated, with Iss < Iss.c (*P* < 0.005), indicating that these five genes have potential to be used as markers (Table 2).

#### Potential of using mitochondrial 12S and 16S rRNA genes as markers based on clade arrangement and other criteria

The nuclear *18S* rRNA gene showed all four clades to be monophyletic, whereas the *28S* rRNA gene showed two (clades I and IV) of the four clades to be monophyletic (Table 2 and Additional file 3: Figure S1, Additional file 4: Figure S2). Monophyly of three of the four clades with strong bootstrap (BS) and posterior probability (PP) support was observed for mitochondrial *cox*1 and *12S* and *16S* rRNA genes, with clade III being not monophyletic (Table 2; Additional file 5: Figures S3, Additional file 6: Figure S4, Additional file 7: Figure S5). Both ML and BI phylogenetic analyzes provided similar tree topologies and clade arrangements. However, despite the good resolution and strong BS and PP support for the *18S* rRNA gene, a higher frequency of indels was present for the *18S* rRNA sequences compared with mitochondrial sequences. A lower frequency of indels enabled a more straightforward sequence alignment when using mitochondrial genes. Moreover, the majority of the *18S* rRNA gene sequences available in NCBI are partial, whereas full-length mitochondrial gene sequences are readily available.

The five genetic markers were evaluated based on four criteria, and the results show that the mitochondrial genes provided better resolution than the nuclear genes for the selected nematode species. Furthermore, the evaluation showed that mitochondrial *12S* and *16S* rRNA genes are comparable to *cox*1 and provide better resolution than the nuclear *28S* rRNA gene (Table 2). Mitochondrial *12S* and *16S* rRNA genes may be used as alternative genetic markers to the nuclear *18S* rRNA gene for nematode systematics.

### The utility of mitochondrial *12S* and *16S* rRNA genes for nematode molecular systematics

#### Phylogeny and genetic distance of nematodes obtained from 12S mitochondrial rRNA gene

**Fig. 1:**
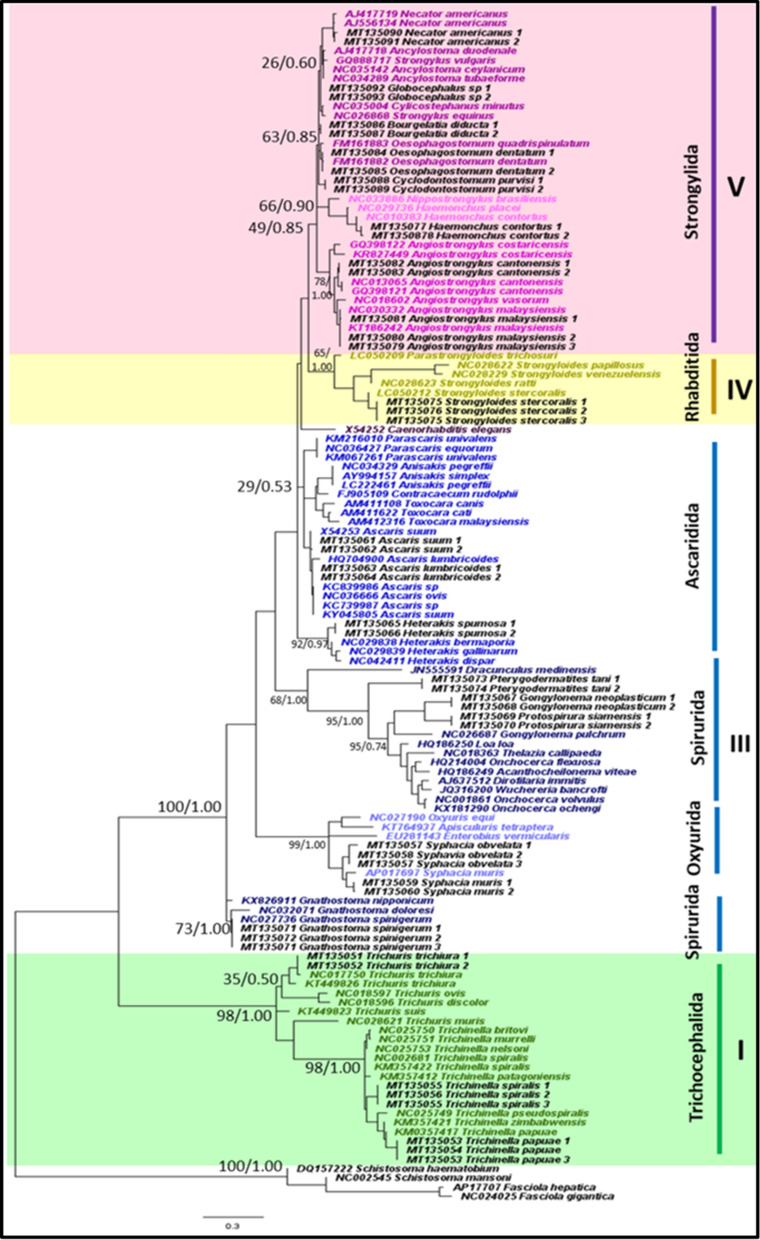
Phylogeny of representative species using mitochondrial *12S* rRNA gene sequences as a genetic marker. Phylogenetic analyzes using maximum likelihood (HKY + G) and Bayesian inference. Numbers at nodes indicate bootstrap values from ML analysis and posterior probability values from BI analysis (ML/BI). Different colors correspond to each nematode clade: outgroup (black); clade I (green); clade III (blue); clade IV (yellow); clade V (purple); and sequences generated in this study (black). Monophyletic clades are highlighted in their respective color

Of the 21 representative species that were sampled in this study, all nematode species were accurately identified to species level using the mitochondrial *12S* rRNA gene and appropriately placed on both ML and BI trees (Fig. 1). Tree topologies and clade arrangements for ML and BI analyzes were similar, and the monophyly of clades I, IV, and V was found, except for *Caenorhabditis elegans* and clade III. PP values for BI analyzes were higher for monophyletic clades compared to BS values from ML analysis. Tree topology supported the close relationship between clades IV and V, which was further substantiated by the two clades having the smallest p-distance value of 21.2%, as shown in Table 3. Within clade III, nematodes belonging to order Ascaridida and Spirurida were not monophyletic, whereas Oxyurida was monophyletic with strong BS and PP support. Within Ascaridida, superfamily Ascaridoidea was a sister clade to the Strongylida and Rhabditida clade, whereas the superfamily Heterakoidea was a sister clade to the Ascaridoidea, Strongylida and Rhabidtida clade. Within Spirurida, the superfamily Gnathostomatoidea was not grouped with the remainder of Spirurida but showed a close relationship with Oxyurida.

**Table 3:** Percentage nucleotide difference between nematode clades based on mitochondrial *12S* rRNA gene expressed in percentage

Clade	I	III	IV	V
I				
III	45.2			
IV	43.7	26.4		
V	40.6	21.3	21.2	
Outgroup	50.0	51.3	53.7	51.1

#### Phylogeny and genetic distance of nematodes obtained from mitochondrial *16S* rRNA gene

**Fig. 2:**
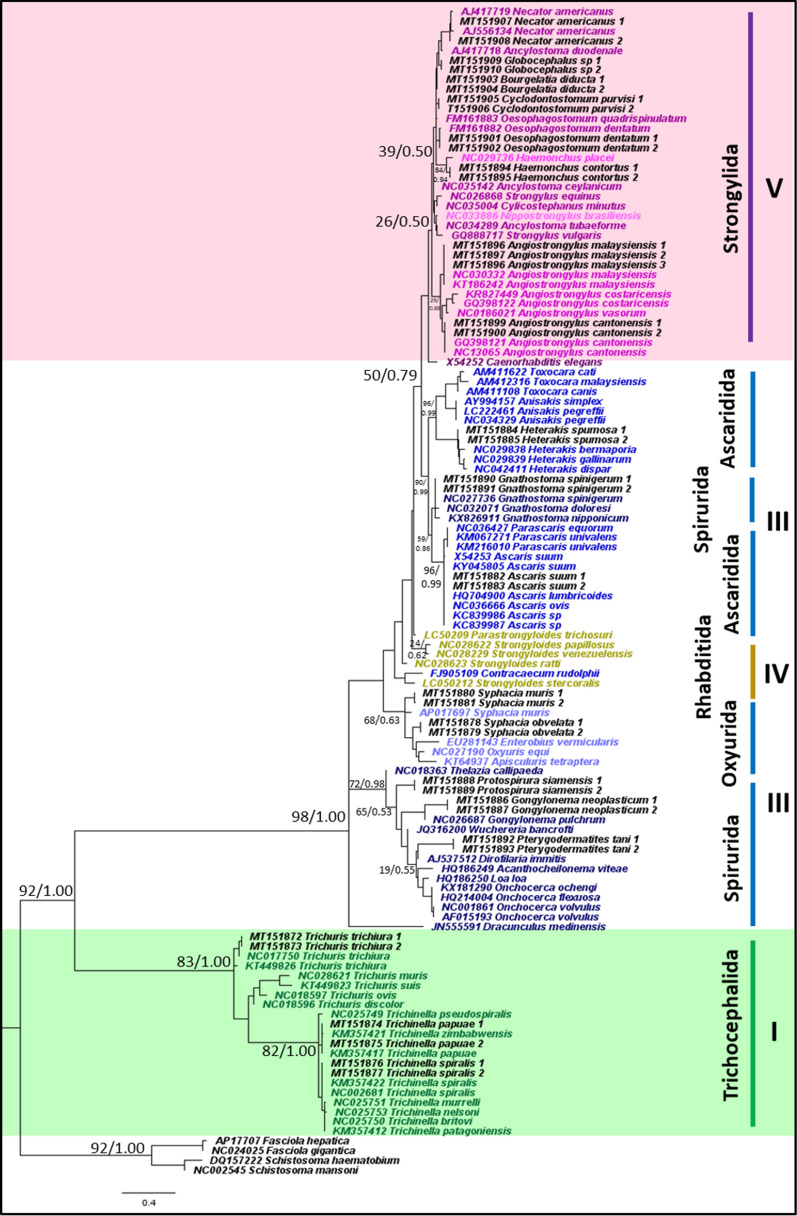
Phylogeny of representative species using mitochondrial *16S* rRNA gene sequences as a genetic marker. Phylogenetic analyzes using maximum likelihood (HKY + G) and Bayesian inference. Numbers at nodes indicate bootstrap values from ML analysis and posterior probability values from BI analysis (ML/BI). Different colors correspond to each nematode clade: outgroup (black); clade I (green); clade III (blue); clade IV (yellow); clade V (purple); and sequences generated in this study (black). Monophyletic clades are highlighted in their respective color

Similarly, of the 21 representative species that were sampled in this study, the mitochondrial *16S* rRNA gene was successfully amplified from all nematode species, and the gene was used to accurately identify them to species level and appropriately place them on the phylogenetic trees. The tree topologies and clade arrangements for ML and BI analyzes were similar. Monophyly of clades I and V were obtained, with strong support for clade I and weak support for clade V (Fig. 2). Based on genetic distance, clades IV and V had the closest relationship, with the smallest p-distance value of 19.2%, as shown in Table 4. Clade III was not monophyletic, which is similar to the tree phylogeny using the mitochondrial *12S* rRNA gene. The Gnathostomatoidea was nested in between the Ascaridida, instead of being closer to the rest of the Spirurida. The clade containing Ascaridida and Gnathostomatoidea was a sister clade to Strongylida; however, support was moderate, with a BS value of 50 and PP of 0.79. Support for the monophyletic clade of Oxyurida and the rest of Spirurida (except Gnathostomatoidea and *Dracunculus mediensis*) was also moderate. Also, clade IV, consisting of the genera *Strongyloides* and *Parastrongyloides*, was not monophyletic.

**Table 4:** Percentage nucleotide difference between nematode clades based on mitochondrial *16S* rRNA gene expressed in percentage

Clade	I	III	IV	V
I				
III	46.2			
IV	44.4	25.4		
V	44.3	23.5	19.2	
Outgroup	45.6	47.9	49.3	47.6

#### Phylogeny and genetic distance of nematodes obtained with concatenated mitochondrial *12S* and *16S* rRNA genes

**Fig. 3:**
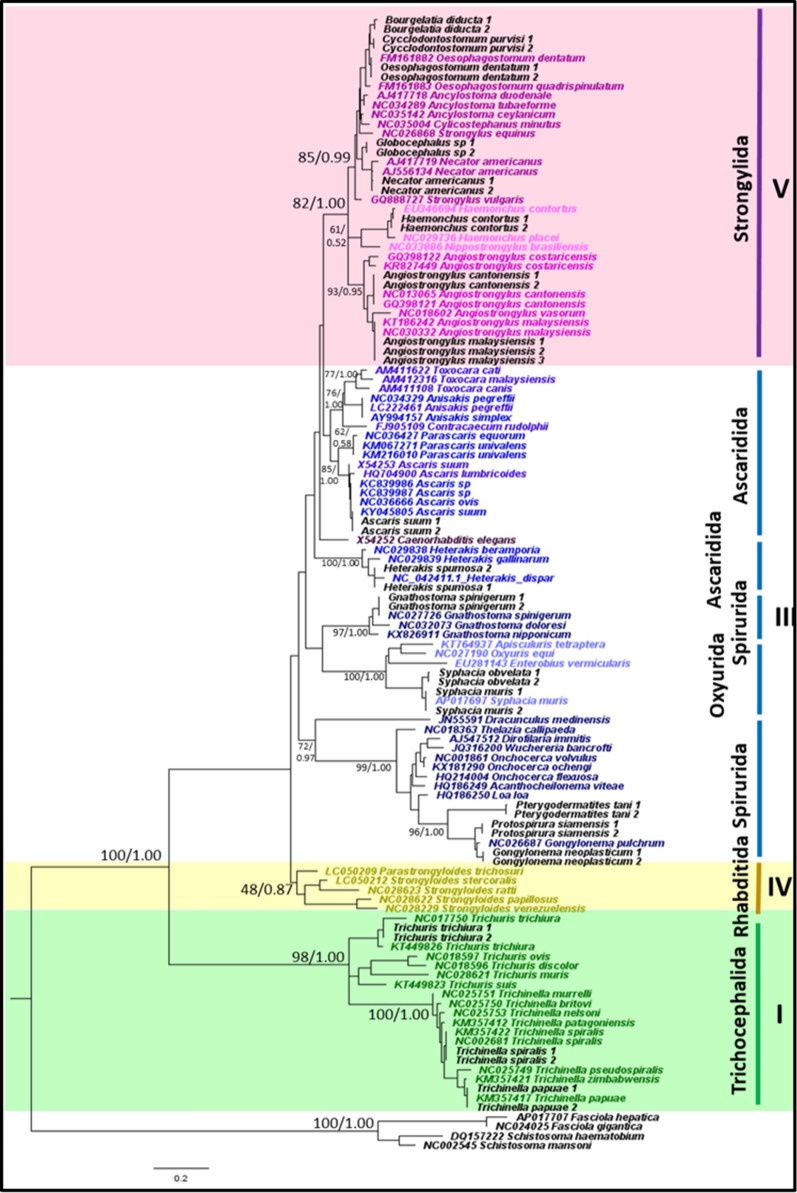
Phylogeny of representative species using mitochondrial concatenated *12S* and *16S* rRNA sequences as a genetic marker. Phylogenetic analyzes using maximum likelihood (HKY + G) and Bayesian inference. Numbers at nodes indicate bootstrap values from ML analysis and posterior probability values from BI analysis (ML/BI). Different colors correspond to each nematode clade: outgroup (black); clade I (green); clade III (blue); clade IV (yellow); clade V (purple); and sequences generated in this study (black). Monophyletic clades are highlighted in their respective color

The phylogeny resulting from the concatenated mitochondrial *12S* and *16S* rRNA genes showed monophyly in clades I, IV and V, and *C. elegans* was not placed together with clade V, which is similar to that seen in the *12S* rRNA gene phylogeny (Fig. 3). Compared to the *12S* rRNA gene, support for clade IV was less robust. Clade IV was placed as a sister clade to the remainder of the species (except clade I), whereas, in *12S* rRNA gene phylogeny, clade IV was a sister clade to clade V. P-distance values showed that clade III and V had the closest relationship, with a p-distance value of 25.1% (Table 5), which is in contrast to the results obtained from *12S* and *16S* rRNA gene analyzes. Likewise, according to *12S* and *16S* rRNA gene phylogenies, clade III was not monophyletic. Within clade III, Ascaridida and Spirurida were not monophyletic, while Oxyurida was monophyletic with strong BS and PP support. Within Ascaridida, the superfamily Ascaridoidea showed a close relationship to Strongylida, while Heterakoidea was a sister clade to the Ascaridoidea and Strongylida clade. As with *12S* rRNA gene phylogeny, the superfamily Gnathostomatoidea was a sister clade to Oxyurida.

**Table 5:** Percentage nucleotide difference between nematode clades based on concatenated mitochondrial *12S* and *16S* rRNA genes expressed in percentage

Clade	I	III	IV	V
I				
III	49.8			
IV	47.6	28.0		
V	44.2	25.1	25.8	
Outgroup	56.8	55.7	55.8	52.8

## Discussion

We have demonstrated the potential of using mitochondrial *12S* and *16S* rRNA genes as genetic markers for molecular systematics of parasitic nematodes through comparisons with other commonly used genetic markers. We have also evaluated their utility through phylum-wide phylogenetic analyzes, providing evidence of their value as robust genetic markers for the study of nematode relationships.

### Potential utility of mitochondrial *12S* rRNA gene in nematode molecular systematics

Phylogenetic relationships were inferred using 120 sequences from 75 species of nematodes, showing the potential of the *12S* rRNA gene as an effective marker for nematode molecular systematics. First, clades I, IV and V were monophyletic in our analyzes, revealing that this genetic marker has sufficient resolution to discriminate between the clades. Although clade III being not monophyletic, our results showed a congruence with previous studies that used whole mitochondrial genomes, in which clade III was not monophyletic [8–10]. The non-monophyly of clade III, consisting of orders Ascaridida, Spirurida and Oxyurida, remains unresolved. However, this demonstrates that the *12S* rRNA gene is robust for molecular systematics, provides sufficient resolution, and is comparable to the whole mitochondrial genome. Even though the partial *12S* and *16S* rRNA sequences are only 460 bp and 240 bp in length, respectively, amplification of a short-length genetic target to study nematode phylogeny is undoubtedly more useful and practical than sequencing whole genomes. For example, long-preserved specimens which may be affected by genetic degeneration issue (e.g. DNA template shearing or fragmentation), could benefit from short-length genetic amplification.

Secondly, mitochondrial *12S* rRNA showed that there was a close relationship between clades IV and V, which has also been found in studies using the *18S* rRNA gene and the transcriptome [7, 11, 12, 30]. However, compared to phylogenetic studies using whole mitochondrial genomes, clade V was more closely related to Ascaridida, whereas clade IV was a sister clade to the clade consisting of clade V and Ascaridida [8–10]. *Strongyloides* and *Parastrongyloides*, the clade IV representative species used in this study, have conventionally been classified to the order Rhabditida, with a close relationship to clade V nematodes. However, they seem to be distantly related to other members of clade IV based on morphology. However, studies using molecular techniques have revealed a different phylogenetic position for *Strongyloides*. Using whole mitochondrial genomes, Lv et al. [31] revealed that *Strongyloides* is nested within clade III nematodes, and the authors also suggested that clade IV may be paraphyletic.

Thirdly, because the *12S* rRNA gene showed clade IV to be monophyletic, it has greater discriminatory potential than *16S* rRNA. The *12S* rRNA gene provided sufficient discrimination between clades in terms of molecular systematics for nematodes, with monophyly found for three of the four clades. Even though the tree topology from concatenated *12S* and *16S* rRNA gene analysis also resulted in monophyly for three of the four clades, sufficient resolution was gained from the use of a single gene; thus, the use of concatenated genes is unnecessary.

Lastly, the sensitivity and specificity of the primers was shown through amplification of a single larva. This enables a convenient and robust method for the study of molecular systematic. Taken together, the advantages of utilizing the *12S* rRNA gene as a genetic marker for molecular systematics of nematodes were demonstrated through the ease of its application and its congruence with phylogenies from whole mitochondrial genomes, as well as from the nuclear *18S* rRNA gene.

### Non-monophyly of clade III nematodes and phylogenetic position of *Gnathostoma*

Clade III, containing orders Ascaridida, Spirurida and Oxyurida, was not found to be monophyletic in any of our analyzes. In current studies of nematode phylogenies, mitochondrial genes have shown the nematode clade III not to be monophyletic, whereas, the working hypothesis of *18S* rRNA gene phylogenies is that clade III is monophyletic. However, Nadler et al. [32] showed that clade III was not monophyletic by using the *18S* rRNA gene due to the inclusion of the superfamily Gnathostomatoidea. Phylogenies from another study that used the *18S* rRNA gene focused on nematodes belonging to the Spirurida; in addition, the Gnathostomatoidea were not placed with the rest of the Spirurida but instead formed a clade at the basal position of the phylogenetic tree [33]. Similarly, Liu et al. [34] could not resolve the phylogenetic position of *Gnathostoma* using mitochondrial genomes. The authors used the complete mitochondrial genomes of 58 species of nematodes and found that *Gnathostoma* was closely related to Ascaridida but was not placed with the rest of Spirurida. Despite many studies and the use of various genetic markers attempting to resolve these discrepancies, the phylogenetic position of clade III nematodes remains inconclusive. A possible explanation is that the phylogenetic position of *Gnathostoma* is based on the traditional classification using morphological characteristics. *Gnathostoma* belongs to order Spirurida and is the type-genus of the superfamily Gnathostomatoidea. Nematodes belonging to Spirurida have a bilaterally symmetrical anterior extremity, the lateral external labial papillae are absent, and they mostly dwell in the tissues of the host [5, 6]. However, when the phylogenetic position of *Gnathostoma* was determined based on molecular genetic markers, an incongruence between morphological and molecular phylogenies was revealed. We propose the inclusion of *Gnathostoma* into future nematode phylogenies for a more accurate picture of its taxonomy. A more comprehensive study with a focus on only clade III nematodes or the inclusion of a greater number of taxa may provide more clarity of nematode phylogenies.

### Advantage of mitochondrial *12S* and *16S* rRNA over nuclear *18S* rRNA gene

The current working hypothesis, first proposed by Blaxter et al. [7], for the molecular systematics of nematodes used *18S* rRNA gene-based phylogenies. However, recently, mitochondrial genomes have served as alternative genetic markers for determining nematode relationships [8–10]. Mitochondrial DNA has a faster evolutionary rate compared to nuclear ribosomal genes such as *18S* rRNA, and this confers an advantage to mitochondrial genes because of their ability to discriminate closely related species [13–15]. Several studies using the nuclear *18S* rRNA gene were unable to provide taxonomic clarity at the species level, as there was insufficient sequence variation between species. In the closely related and morphologically similar *Haemonchus placei* and *H. contortus*, the nuclear *18S* rRNA gene sequences are identical [35–37]. Among *Trichuris* spp., morphological variations also confound morphological identification [38, 39]. Moreover, the highly similar nuclear *18S* rRNA sequences of *T. vulpis* and *T. serrata* indicate that this gene might not have sufficient sequence variation to discriminate between closely related species [39].

In this study, we demonstrated the resolution power of mitochondrial *12S* and *16S* rRNA genes for species discrimination through the phylogenetic placements of nematode species using these two genetic markers. Despite the nuclear *18S* rRNA gene provides better resolution at higher taxonomic levels because of the sufficient variation between clades, as seen in monophyletic clades, the gene’s resolution at lower taxonomic levels remains ambiguous, as seen in the species discrimination results. Undoubtedly, mitochondrial *12S* and *16S* rRNA genes have advantages over nuclear *18S* rRNA as phylogenetic markers. They provided a better resolution at lower taxonomic levels because of sufficient genetic variation between species, as suggested by the phylogenetic placement of nematode species in our analyzes, as well as a resolution at higher taxonomic levels as a result of sufficient genetic variation between clades, except for clade III, as indicated by monophyly. Thus, utilizing mitochondrial *12S* and *16S* rRNA genes as genetic markers for nematode molecular systematics may not only provide resolution at lower taxonomic levels but also higher levels across a wide-ranging taxonomic hierarchy.

### Potential use of mitochondrial *12S* and *16S* rRNA genes for nematode DNA barcoding

A DNA barcode is defined as a short sequence of DNA that can accurately identify organisms [40–42]. The concept of DNA barcoding was first proposed by Herbert et al. [42] when they suggested using DNA sequences as taxon ‘barcodes’ for the global identification of all organisms. Species are usually identified by comparing unknown sequences against known reference sequences. Currently, the universal genetic marker used for DNA barcoding is the 5ʹ-end of the mitochondrial *cox*1 gene. The *cox*1 gene has specific properties that make it useful as a genetic marker for DNA barcoding, including multiple copies, robust PCR amplification, nearly identical sequences in individuals of the same species yet sufficient variation between species, and the presence of a reference database for sequence comparison [40–42]. In this study, the viability of using mitochondrial *12S* and *16S* rRNA genes for DNA barcode identification of nematodes species was demonstrated; 100% of our representative nematodes species were accurately taxonomically assigned, suggesting that both genes have sufficient genetic variation to allow for species identification. Furthermore, as both *12S* and *16S* rRNA genes are mitochondrial in origin, PCR amplification success and robustness will be comparable to the *cox*1 gene. The mitochondrial *12S* and *16S* rRNA genes contain more highly conserved sequences than *cox*1, facilitating easier primer design, but also contain sufficient genetic variation for species identification.

Although primers had to be designed separately for nematodes of clade I and nematodes of clades III, IV and V, the same region was used for primer design. This enabled DNA sequence and phylogenetic analyzes of the clades to be performed together, maximizing the range of taxa studied. Although the current universal gene for DNA barcoding studies of animals is *cox*1, an alternative gene for species identification could be useful. Studies have evaluated the potential use of the mitochondrial *16S* rRNA gene as a DNA barcode for insects [43, 44]. A comparison between mitochondrial *cox*1 and *16S* rRNA gene was performed, and the authors concluded that the *16S* rRNA gene could detect more taxa than *cox*1 because of reduced PCR amplification bias.

Ferri et al. [45] compared the performance of mitochondrial *cox*1 and *12S* rRNA genes for DNA barcoding of Spirurida nematodes and suggested that the *12S* rRNA gene could be appropriate for species-level identification. However, in order for mitochondrial *12S* and *16S* rRNA genes to be utilized in DNA barcoding, it is essential to establish sufficient sequences in the reference databases. The advantage of the *cox*1 gene over other genetic markers, such as mitochondrial *12S* and *16S* rRNA genes, is the extensive availability of database sequences, allowing for thorough comparisons of unknown sequences. However, the potential of mitochondrial *12S* and *16S* rRNA genes for DNA barcoding of nematodes cannot be disregarded, as we have shown the capability of these two genetic markers to discriminate between species. Therefore, mitochondrial ribosomal genes have considerable value, not only as markers for molecular systematics studies but also their potential use in DNA barcoding studies. Future studies are needed to evaluate further the potential and utility of mitochondrial rRNA genes for DNA barcoding and to expand the reference databases for these genetic markers.

## Limitations

Clade II and plant-parasitic nematodes belonging to clade IV were not included in this study. The exclusion of representative species from these two groups could have resulted in an incomplete picture of phylum-wide relationships. Moreover, the primers designed in this study are clade-specific because it was challenging to find regions conserved across the four clades. Primers had to be designed separately for nematodes belonging to clade I and nematodes belonging to clades III, IV and V. An increase in the number of taxa could potentially aid in the generation of a complete picture of phylum-wide relationships.

## Conclusions

We evaluated the potential of using mitochondrial *12S* and *16S* rRNA genes as phylogenetic markers for molecular systematics by comparing them with other commonly used markers. Through phylogenetic analyzes of 75 nematode taxa, we have shown that the *12S* rRNA gene could be an excellent genetic marker, providing robust and comparable phylogenies. We demonstrated the resolution power of mitochondrial *12S* and *16S* rRNA genes at both lower and higher taxonomic levels for species and clade discrimination, and we showed they are potent markers for molecular systematics of nematodes. In addition, we also demonstrated the benefits of using these two markers for DNA barcoding studies. Future research perspectives include an expansion of taxa studied and the use of a combination of different genetic markers from both mitochondrial and nuclear DNA, which may benefit future studies on nematode phylogenetic relationships.

## Supplementary information

**Additional file 1: Table S1.** NCBI sequences used for each genetic marker for phylogenetic analysis.

**Additional file 2: Table S2.** NCBI sequences used for *12S* and *16S* rRNA gene primer design.

**Additional file 3: Figure S1.** Phylogeny using *18S* rRNA gene sequences as a genetic marker. Phylogenetic analyzes using maximum likelihood (TN93 + G) and Bayesian inference. Numbers at nodes indicate bootstrap values from ML analysis and posterior probability values from BI analysis (ML/BI). Different colors correspond to each nematode clade: outgroup (black); clade I (green); clade III (blue); clade IV (yellow); and clade V (purple). Monophyletic clades are highlighted in their respective color.

**Additional file 4: Figure S2.** Phylogeny using *28S* rRNA gene sequences as a genetic marker. Phylogenetic analyzes using maximum likelihood (TN93 + G) and Bayesian inference. Numbers at nodes indicate bootstrap values from ML analysis and posterior probability values from BI analysis (ML/BI). Different colors correspond to each nematode clade: outgroup (black); clade I (green); clade III (blue); clade IV (yellow); and clade V (purple). Monophyletic clades are highlighted in their respective color.

**Additional file 5: Figure S3.** Phylogeny using *cox*1 gene sequences as a genetic marker. Phylogenetic analyzes using maximum likelihood (GTR + G) and Bayesian inference. Numbers at nodes indicate bootstrap values from ML analysis and posterior probability values from BI analysis (ML/BI). Different colors correspond to each nematode clade: outgroup (black); clade I (green); clade III (blue); clade IV (yellow); clade V (purple). Monophyletic clades are highlighted in their respective color.

**Additional file 6: Figure S4.** Phylogeny using *12S* rRNA gene sequences as a genetic marker. Phylogenetic analyzes using maximum likelihood (HKY + G) and Bayesian inference. Numbers at nodes indicate bootstrap values from ML analysis and posterior probability values from BI analysis (ML/BI). Different colors correspond to each nematode clade: outgroup (black); clade I (green); clade III (blue); clade IV (yellow); and clade V (purple). Monophyletic clades are highlighted in their respective color.

**Additional file 7: Figure S5.** Phylogeny using *16S* rRNA gene sequences as a genetic marker. Phylogenetic analyzes using maximum likelihood (GTR + G) and Bayesian inference. Numbers at nodes indicate bootstrap values from ML analysis and posterior probability values from BI analysis (ML/BI). Different colors correspond to each nematode clade: outgroup (black); clade I (green); clade III (blue); clade IV (yellow); and clade V (purple). Monophyletic clades are highlighted in their respective color.

## Data Availability

All data generated during this study are included in the published article and its additional files. The newly generated sequences were deposited in the GenBank database under the accession numbers MT135051–MT135093 and MT151872–MT151910.

## Abbreviations

WHO: World Health Organization
PCR: polymerase chain reaction
NCBI: National Center for Biotechnology Information
*cox*1: cytochrome *c* oxidase subunit 1
ITS1: internal transcribed spacer 1
ITS2: internal transcribed spacer 2
rRNA: ribosomal RNA
ML: maximum likelihood
BI: Bayesian inference
BS: bootstrap
PP: posterior probability
