# Supplementary material for: Evaluation and utility of mitochondrial ribosomal genes for molecular systematics of parasitic nematodes
Source: Parasit Vectors. 2020 Jul 20;13:364. doi: 10.1186/s13071-020-04242-8 (PMC7372814; doi:10.1186/s13071-020-04242-8)
Supplement: Supplementary file 1 — Additional file 1: Table S1. NCBI sequences used for each genetic marker for phylogenetic analysis. [file 13071_2020_4242_MOESM1_ESM.docx]

**Additional file 1: Table S1.** NCBI sequences used for each genetic marker for phylogenetic analysis.

| Genetic marker | Clade | Superfamily | Species | NCBI accession number |
| --- | --- | --- | --- | --- |
| *12S* rRNA  *16S* rRNA  *cox1* gene | I | Trichinelloidea | *Trichuris muris* | NC028621 |
|  |  |  | *Trichuris ovis* | NC018597 |
|  |  |  | *Trichuris discolor* | NC018596 |
|  |  |  | *Trichuris trichiura* | NC017750 |
|  |  |  | *Trichuris trichiura* | KT449826 |
|  |  |  | ***Trichuris trichiura*** | **MT135051/ MT151872** |
|  |  |  | ***Trichuris trichiura*** | **MT135052/ MT151873** |
|  |  |  | *Trichuris suis* | KT449823 |
|  |  |  | *Trichinella papuae* | KM357417 |
|  |  |  | ***Trichinella papuae*** | **MT135053/ MT151874** |
|  |  |  | ***Trichinella papuae*** | **MT135054/ MT151875** |
|  |  |  | *Trichinella spiralis* | KM357422 |
|  |  |  | *Trichinella spiralis* | NC002681 |
|  |  |  | ***Trichinella spiralis*** | **MT135055/ MT151876** |
|  |  |  | ***Trichinella spiralis*** | **MT135056/ MT151877** |
|  |  |  | *Trichinella zimbabwensis* | KM357421 |
|  |  |  | *Trichinella pseudospiralis* | NC025749 |
|  |  |  | *Trichinella britovi* | NC025750 |
|  |  |  | *Trichinella murrelli* | NC025751 |
|  |  |  | *Trichinella nelsoni* | NC025753 |
|  |  |  | *Trichinella patagoniensis* | KM357412 |
|  | III | Oxyuroidea | *Enterobius vermicularis* | EU281143 |
|  |  |  | *Oxyuris equi* | NC027190 |
|  |  |  | ***Syphacia obvelata*** | **MT135057/ MT151878** |
|  |  |  | ***Syphacia obvelata*** | **MT135058/ MT151879** |
|  |  |  | *Syphacia muris* | AP017697 |
|  |  |  | ***Syphacia muris*** | **MT135059/ MT151880** |
|  |  |  | ***Syphacia muris*** | **MT135060/ MT151881** |
|  |  |  | *Aspiculuris tetraptera* | KT764937 |
|  |  | Ascaridoidea | *Ascaris suum* | X54253 |
|  |  |  | *Ascaris suum* | KY045805 |
|  |  |  | ***Ascaris suum*** | **MT135061/ MT151882** |
|  |  |  | ***Ascaris suum*** | **MT135062/ MT151883** |
|  |  |  | *Ascaris ovis* | NC036666 |
|  |  |  | *Ascaris lumbricoides* | HQ704900 |
|  |  |  | ***Ascaris lumbricoides*** | **MT135063** |
|  |  |  | ***Ascaris lumbricoides*** | **MT135064** |
|  |  |  | *Ascaris sp.* | KC839986 |
|  |  |  | *Ascaris sp.* | KC839987 |
|  |  |  | *Parascaris univalens* | KM216010 |
|  |  |  | *Parascaris univalens* | KM067271 |
|  |  |  | *Parascaris equorum* | NC036427 |
|  |  |  | *Toxocara cati* | AM411622 |
|  |  |  | *Toxocara canis* | AM411108 |
|  |  |  | *Toxocara malaysiensis* | AM412316 |
|  |  |  | *Anisakis pegreffii* | LC222461 |
|  |  |  | *Anisakis pegreffii* | NC034329 |
|  |  |  | *Anisakis simplex* | AY994157 |
|  |  |  | *Contracaecum rudolphii* | FJ905109 |
|  |  | Heterakoidea | *Heterakis gallinarum* | NC029839 |
|  |  |  | *Heterakis beramporia* | NC029838 |
|  |  |  | *Heterakis dispar* | NC042411 |
|  |  |  | ***Heterkis spumosa*** | **MT135065/ MT151884** |
|  |  |  | ***Heterakis spumosa*** | **MT135066/ MT151885** |
|  |  | Filaroidea | *Onchocerca volvulus* | NC001861 |
|  |  |  | *Onchocerca ochengi* | KX181290 |
|  |  |  | *Onchocerca flexuosa* | HQ214004 |
|  |  |  | *Loa loa* | HQ186250 |
|  |  |  | *Wuchereria bancrofti* | JQ316200 |
|  |  |  | *Acanthocheilonema viteae* | HQ186249 |
|  |  |  | *Dirofilaria immitis* | AJ537512 |
|  |  | Thelazoidea | *Thelazia callipaeda* | NC018363 |
|  |  | Dracunculoidea | *Dracunculas medinensis* | JN555591 |
|  |  | Spiruroidea | *Gongylonema pulchrum* | NC026687 |
|  |  |  | ***Gongylonema neoplasticum*** | **MT135067/ MT151886** |
|  |  |  | ***Gongylonema neoplasticum*** | **MT135068/ MT151887** |
|  |  |  | ***Protospirura siamensis*** | **MT135069/ MT151888** |
|  |  |  | ***Protospirura siamensis*** | **MT135070/ MT151889** |
|  |  | Gnathostomatoidea | *Gnathostoma nipponicum* | KX826911 |
|  |  |  | *Gnathostoma doloresi* | NC032073 |
|  |  |  | *Gnathostoma spinigerum* | NC027726 |
|  |  |  | ***Gnathostoma spinigerum*** | **MT135071/ MT151890** |
|  |  |  | ***Gnathostoma spinigerum*** | **MT135072/ MT151891** |
|  |  | Rictularoidea | ***Pterygodermatites tani*** | **MT135073/ MT151892** |
|  |  |  | ***Pterygodermatites tani*** | **MT135074/ MT151893** |
|  | IV | Rhabdiasoidea | *Strongyloides papillosus* | NC028622 |
|  |  |  | *Strongyloides ratti* | NC028623 |
|  |  |  | *Strongyloides venezuelensis* | NC028229 |
|  |  |  | *Strongyloides stercoralis* | LC050212 |
|  |  |  | ***Strongyloides stercoralis*** | **MT135075** |
|  |  |  | ***Strongyloides stercoralis*** | **MT135076** |
|  |  |  | *Parastrongyloides trichosuri* | LC050209 |
|  | V | Trichostrongyloidea | *Haemonchus placei* | NC029736 |
|  |  |  | *Haemonchus contortus* | EU346694 |
|  |  |  | ***Haemonchus contortus*** | **MT135077/ MT151894** |
|  |  |  | ***Haemonchus contortus*** | **MT135078/ MT151895** |
|  |  |  | *Nippostrongylus brasiliensis* | NC033886 |
|  |  | Metastrongyloidea | *Angiostrongylus malaysiensis* | NC030332 |
|  |  |  | *Angiostrongylus malaysiensis* | KT186242 |
|  |  |  | ***Angiostrongylus malaysiensis*** | **MT135079/ MT151896** |
|  |  |  | ***Angiostrongylus malaysiensis*** | **MT135080/ MT151897** |
|  |  |  | ***Angiostrongylus malaysiensis*** | **MT135081/ MT151898** |
|  |  |  | *Angiostrongylus cantonensis* | GQ398121 |
|  |  |  | *Angiostrongylus cantonensis* | NC013065 |
|  |  |  | ***Angiostrongylus cantonensis*** | **MT135082/ MT151899** |
|  |  |  | ***Angiostrongylus cantonensis*** | **MT135083/ MT151900** |
|  |  |  | *Angiostrongylus costaricensis* | GQ398122 |
|  |  |  | *Angiostrongylus costaricensis* | KR827449 |
|  |  |  | *Angiostrongylus vasorum* | NC018602 |
|  |  | Strongyloidea | *Oesophagostomum quadrispinulatum* | FM161883 |
|  |  |  | *Oesophagostomum dentatum* | FM161882 |
|  |  |  | ***Oesophagostomum dentatum*** | **MT135084/ MT151901** |
|  |  |  | ***Oesophagostomum dentatum*** | **MT135085/ MT151902** |
|  |  |  | ***Bourgelatia diducta*** | **MT135086/ MT151903** |
|  |  |  | ***Bourgelatia diducta*** | **MT135087/ MT151904** |
|  |  |  | ***Cyclodontostomum purvisi*** | **MT135088/ MT151905** |
|  |  |  | ***Cyclodontostomum purvisi*** | **MT135089/ MT151906** |
|  |  |  | *Strongylus vulgaris* | GQ888717 |
|  |  |  | *Strongylus equinus* | NC026868 |
|  |  |  | *Cylicostephanus minutus* | NC035004 |
|  |  | Ancylostomatoidea | *Necator americanus* | AJ417719 |
|  |  |  | *Necator americanus* | AJ556134 |
|  |  |  | ***Necator americanus*** | **MT135090/ MT151907** |
|  |  |  | ***Necator americanus*** | **MT135091/ MT151908** |
|  |  |  | ***Globocephalus sp.*** | **MT135092/ MT151909** |
|  |  |  | ***Globocephalus sp.*** | **MT135093/ MT151910** |
|  |  |  | *Ancylostoma duodenale* | AJ417718 |
|  |  |  | *Ancylostoma ceylanicum* | NC035142 |
|  |  |  | *Ancylostoma tubaeforme* | NC034289 |
|  |  | Rhabditoidea | *Caenorhabditis elegans* | X54252 |
|  | Outgroup | Echinostomatoidea | *Fasciola hepatica* | AP017707 |
|  |  |  | *Fasciola gigantica* | NC024025 |
|  |  | Schistosomatoidea | *Schistosoma haematobium* | DQ157222 |
|  |  |  | *Schistosoma mansoni* | NC002545 |
| *ITS1* | I | Trichinelloidea | *Trichinella spiralis* | KU374868 |
|  |  |  | *Trichinella spiralis* | KC006423** |
|  |  |  | *Trichinella spiralis* | KC006422 |
|  |  |  | *Trichuris trichiura* | GQ301555 |
|  |  |  | *Trichuris trichiura* | KJ588107 |
|  |  |  | *Trichuris trichiura* | KC877992 |
|  | III | Oxyuroidea | *Enterobius vermicularis* | HQ646164** |
|  |  |  | *Syphacia obvelata* | EF464554** |
|  |  | Ascaridoidea | *Ascaris lumbricoides* | GQ339800 |
|  |  |  | *Ascaris lumbricoides* | MF358963 |
|  |  |  | *Ascaris lumbricoides* | GQ339801 |
|  |  | Ascaridoidea | *Anisakis simplex* | KF512906** |
|  |  |  | *Anisakis simplex* | GU735489 |
|  |  |  | *Anisakis simplex* | JX237373 |
|  |  |  | *Toxocara canis* | JF837169 |
|  |  |  | *Toxocara cati* | JF837173** |
|  |  |  | *Toxocara vitulorum* | KY442062 |
|  |  | Heterakoidea | *Heterakis spumosa* | JX845278** |
|  |  | Spiruoidea | *Gongylonema pulchrum* | AB495401** |
|  |  |  | *Gongylonema neoplasticum* | LC331000 |
|  |  |  | *Gongylonema nepalensis* | LC388743 |
|  |  | Filaroidea | *Dirofilaria immitis* | AF217800** |
|  |  |  | *Dirofilaria immitis* | EU087700 |
|  |  |  | *Wuchereria bancrofti* | EU272178 |
|  |  |  | *Onchocerca volvulus* | EU272179 |
|  |  |  | *Onchocerca volvulus* | AF228573 |
|  | IV | Rhabdiasoidea | *Strongyloides stercoralis* | JF699148 |
|  |  |  | *Strongyloides stercoralis* | JX489153** |
|  |  |  | *Strongyloides stercoralis* | EF653266** |
|  | V | Trichostrongyloidea | *Haemonchus contortus* | EU084682 |
|  |  |  | *Haemonchus contortus* | KJ938043 |
|  |  | Metastrongyloidea | *Angiostrongylus cantonensis* | GU587760 |
|  |  |  | *Angiostrongylus cantonensis* | KP776413 |
|  |  |  | *Angiostrongylus costaricensis* | GU587746 |
|  |  |  | *Angiostrongylus vasorum* | GU733324 |
|  |  | Strongyloidea | *Oesohagostomum dentatum* | AJ619979 |
|  |  |  | *Oesophagostomum quadrispinulatum* | AJ889567 |
|  |  | Ancylostomatoidea | *Necator americanus* | MH665842 |
|  |  |  | *Necator americanus* | KM891738 |
|  |  |  | *Ancylostoma duodenale* | EU344797 |
|  |  |  | *Ancylostoma duodenale* | AJ001679 |
|  |  |  | *Ancylostoma tubeforme* | KY474053 |
|  |  |  | *Ancylostoma ceylanicum* | KY640230 |
|  | Outgroup | Echinostomatoidea | *Fasciola hepatica* | MG569981 |
|  |  |  | *Fasciola gigantica* | HM746787 |
|  |  | Schistosomatoidea | *Schistosoma haematobium* | GU257398 |
|  |  |  | *Schistosoma mansoni* | KX011042 |
| *ITS2* | I | Trichinelloidea | *Trichinella spiralis* | AY851266 |
|  |  |  | *Trichinella spiralis* | AF342803** |
|  |  |  | *Trichinella spiralis* | KC006423** |
|  |  |  | *Trichuris trichiura* | KJ588164 |
|  |  |  | *Trichuris trichiura* | JN181860 |
|  |  |  | *Trichuris trichiura* | AM992983 |
|  | III | Oxyuroidea | *Enterobius vermicularis* | HQ646164** |
|  |  |  | *Enterobius vermicularis* | MH203027 |
|  |  |  | *Syphacia muris* | MF142453 |
|  |  | Ascaridoidea | *Ascaris lumbricoides* | MF358961 |
|  |  |  | *Ascaris lumbricoides* | AB110029 |
|  |  |  | *Ascaris lumbricoides* | LC422643 |
|  |  |  | *Anisakis simplex* | GQ143711 |
|  |  |  | *Anisakis simplex* | KF512906** |
|  |  |  | *Anisakis simplex* | MF959775 |
|  |  |  | *Toxocara vitulorum* | KY442062 |
|  |  |  | *Toxocara cati* | JF837173** |
|  |  | Heterakoidea | *Heterakis isolonche* | KM212953 |
|  |  |  | *Heterakis spumosa* | JX845278** |
|  |  | Spiruroidea | *Gongylonema pulchrum* | AB495401** |
|  |  |  | *Gongylonema pulchrum* | AB495402 |
|  |  | Filaroidea | *Onchocerca volvulus* | AF228566 |
|  |  |  | *Onchocerca volvulus* | EU272179 |
|  |  |  | *Dirofilaria immitis* | AF217800** |
|  |  |  | *Dirofilaria immitis* | EU087699 |
|  |  |  | *Dirofilaria immitis* | EU182331 |
|  | IV | Rhabdiasoidea | *Strongyloides stercoralis* | JX489153** |
|  |  |  | *Strongyloides stercoralis* | EF653266** |
|  | V | Trichostrongyloidea | *Haemonchus contortus* | EU084691 |
|  |  |  | *Haemonchus contortus* | KX534104 |
|  |  | Metastrongyloidea | *Angiostrongylus cantonensis* | MF371332 |
|  |  |  | *Angiostrongylus cantonensis* | KU528693 |
|  |  |  | *Angiostrongylus cantonensis* | EU636008 |
|  |  | Ancylostomatoidea | *Necator americanus* | MG256601 |
|  |  |  | *Necator americanus* | AF217891 |
|  |  |  | *Necator americanus* | JF960372 |
|  |  |  | *Ancylostoma duodenale* | MK271367 |
|  |  |  | *Ancylostoma duodenale* | MG271919 |
|  |  |  | *Ancylostoma duodenale* | KC632570 |
|  | Outgroup | Echinostomatoidea | *Fasciola hepatica* | AB553733 |
|  |  |  | *Fasciola gigantica* | AB553719 |
|  |  | Schistosomatoidea | *Schistosoma haematobium* | AF146038 |
|  |  |  | *Schistosoma mansoni* | KX011042 |
| *18S* rRNA gene | I | Trichinelloidea | *Trichinella spiralis* | U60231 |
|  |  |  | *Trichinella spiralis* | KU725992 |
|  |  |  | *Trichuris trichuira* | AB699091 |
|  |  |  | *Trichuris trichuira* | GQ352553 |
|  | III | Oxyuroidea | *Enterobius vermicularis* | HQ646164** |
|  |  |  | *Enterobius vermicularis* | JF934731 |
|  |  |  | *Syphacia obvelata* | EF464554** |
|  |  |  | *Syphacia muris* | EF464553 |
|  |  |  | *Oxyuris equi* | EF180062 |
|  |  |  | *Oxyuris equi* | KU180664 |
|  |  | Ascaridoidea | *Ascaris lumbricoides* | U94366 |
|  |  |  | *Ascaris suum* | U94367 |
|  |  |  | *Anisakis sp,* | U94365 |
|  |  |  | *Anisakis simplex* | MF072711 |
|  |  |  | *Anisakis pegreffii* | MF072697 |
|  |  |  | *Toxocara canis* | JN256976 |
|  |  |  | *Toxocara vitulorum* | KJ398347 |
|  |  | Heterakoidea | *Heterakis sp* | AF083003 |
|  |  |  | *Heterakis gallinarum* | DQ503462 |
|  |  |  | *Heterakis spumosa* | MH571872 |
|  |  | Spiruroidea | *Gongylonema pulchrum* | AB495401** |
|  |  | Filaroidea | *Dirofilaria immitis* | MK673810 |
|  |  |  | *Dirofilaria repens* | MK495735 |
|  |  |  | *Wuchereria bancrofti* | AF227234 |
|  |  |  | *Wuchereria bancrofti* | AY843438 |
|  |  |  | *Wuchereria bancrofti* | AY843437 |
|  | IV | Rhabdiasoidea | *Strongyloides stercoralis* | AJ417023 |
|  |  |  | *Strongyloides stercoralis* | M84229 |
|  |  |  | *Strongyloides ratti* | AB453328 |
|  |  |  | *Strongyloides ratti* | AB923889 |
|  |  |  | *Strongyloides ratti* | AB453329 |
|  | V | Trichostronyloidea | *Haemonchus contortus* | EU086375 |
|  |  |  | *Haemonchus sp.* | DQ503465 |
|  |  | Metastrongyloidea | *Angiostrongylus cantonensis* | AY295804 |
|  |  |  | *Angiostrongylus costaricensis* | EF514913 |
|  |  |  | *Angiostrongylus vasorum* | KY654093 |
|  |  |  | *Angiostrongylus malaysiensis* | EF514914 |
|  |  | Strongyloidea | *Oesophagostomum muntiacum* | LC415112 |
|  |  | Ancylostomatoidea | *Necator americanus* | AY295811 |
|  |  |  | *Necator americanus* | AJ920348 |
|  |  |  | *Ancylostoma duodenale* | EU344798 |
|  | Outgroup | Schistosomatoidea | *Schistosoma mansoni* | U65657 |
| 28S rRNA gene | I | Trichinelloidea | *Trichinella spiralis* | AF342803** |
|  | III | Oxyuroidea | *Oxyuris equi* | KU180675 |
|  |  |  | *Oxyuris equi* | KY990021 |
|  |  |  | *Enterobius vermicularis* | LC416069 |
|  |  | Ascaridoidea | *Ascaris suum* | FJ418792 |
|  |  |  | *Ascaris lumbricoides* | AY210806 |
|  |  |  | *Ascaris lumbricoides* | U94751 |
|  |  |  | *Anisakis simplex* | MF094292 |
|  |  |  | *Anisakis pegreffii* | MF094291 |
|  |  |  | *Toxocara vitulorum* | FJ418790 |
|  |  | Heterakoidea | *Heterakis spumosa* | MH571869 |
|  |  | Spiruoidea | *Gongylonema neoplasticum* | LC026033 |
|  |  | Filaroidea | *Onchocerca ochengi* | KP760400 |
|  |  |  | *Dirofilaria immitis* | KY990015 |
|  |  |  | *Dirofilaria immitis* | KP760375 |
|  | IV | Rhabdiasoidea | *Strongyloides stercoralis* | KU180693 |
|  |  |  | *Strongyloides ratti* | U39490 |
|  | V | Trichostrongyloidea | *Haemonchus contortus* | AM039742 |
|  |  | Metastrongyloidea | *Angiostrongylus cantonensis* | AY292792 |
|  |  |  | *Angiostrongylus vasorum* | AM039758 |
|  |  | Ancylostomatoidea | *Necator americanus* | KU180694 |
|  |  |  | *Necator americanus* | AM039740 |
|  |  |  | *Ancylostoma caninum* | AM039739 |
|  |  |  | *Ancylostoma duodenale* | AJ556180 |
|  | Outgroup | Echinostomatoidea | *Fasciola hepatica* | AY222244 |
|  |  |  | *Fasciola gigantica* | AY222245 |
|  |  | Schistosomatoidea | *Schistosoma haematobium* | EU567126 |
|  |  |  | *Schistosoma mansoni* | AY157173 |

** indicates sequences that were used for more than one genetic marker.

NCBI accession numbers in **bold** are the partial *12S* rRNA gene sequences generated in this study, and numbers in **bold** and underlined are the partial *16S* rRNA gene sequences generated in this study.
