# Supplementary material for: Evaluation and utility of mitochondrial ribosomal genes for molecular systematics of parasitic nematodes
Source: Parasit Vectors. 2020 Jul 20;13:364. doi: 10.1186/s13071-020-04242-8 (PMC7372814; doi:10.1186/s13071-020-04242-8)
Supplement: Supplementary file 2 — Additional file 2: Table S2. NCBI sequences used for 12S and 16S rRNA gene primer design. [file 13071_2020_4242_MOESM2_ESM.docx]

**Additional file 2: Table S2.** NCBI sequences used for *12S* and *16S* rRNA gene primer design.

| Accession number | Species | Superfamily | Clade | Primer name |
| --- | --- | --- | --- | --- |
| KM357422 | *Trichinella spiralis* | Trichinelloidea | I | 12S.C1.F  12S.C1.R  16S.C1.F  16S.C1.R |
| NC0257421 | *Trichinella pseudospiralis* |  |  |  |
| KM357417 | *Trichinella papuae* |  |  |  |
| NC017750 | *Trichuris trichiura* |  |  |  |
| NC028621 | *Trichuris muris* |  |  |  |
| KT449823 | *Trichuris suis* |  |  |  |
| NC018596 | *Trichuris ovis* |  |  |  |
| NC001861 | *Onchocerca volvulus* | Filaroidea | III | 12S.C345.F  12S.C345.R  16S.C345.F  16S.C345.R |
| KX181290 | *Onchocerca ochengi* |  |  |  |
| HQ214004 | *Onchocerca flexuosa* |  |  |  |
| HQ186250 | *Loa loa* |  |  |  |
| JQ316200 | *Wuchereria bancrofti* |  |  |  |
| NC018363 | *Thelazia callipaeda* | Thelazoidea |  |  |
| NC026687 | *Gongylonema pulchrum* | Spiruroidea |  |  |
| JN555591 | *Dracunculus medinensis* | Dracunculoidea |  |  |
| NC027190 | *Oxyuris equi* | Oxyuroidea |  |  |
| KT764937 | *Aspiculuris tetraptera* |  |  |  |
| EU281143 | *Enterobius vermicularis* |  |  |  |
| KT900946 | *Syphacia obvelata* |  |  |  |
| NC036666 | *Ascaris ovis* | Ascaridoidea |  |  |
| KY045805 | *Ascaris suum* |  |  |  |
| HQ704900 | *Ascaris lumbricoides* |  |  |  |
| KM216010 | *Parascaris univalens* |  |  |  |
| NC036427 | *Parascaris equorum* |  |  |  |
| AM411622 | *Toxocara cati* |  |  |  |
| AM411108 | *Toxocara canis* |  |  |  |
| AM412316 | *Toxocara malaysiensis* |  |  |  |
| LC222461 | *Anisakis pegreffii* |  |  |  |
| AY994157 | *Anisakis simplex* |  |  |  |
| FJ905109 | *Contracaecum rudolphi* |  |  |  |
| LC050211 | *Strongyloides ratti* | Rhabdiasoidea | IV |  |
| LC050212 | *Strongyloides stercoralis* |  |  |  |
| LC050213 | *Strongyloides venezuelensis* |  |  |  |
| LC050210 | *Strongyloides papillosus* |  |  |  |
| LC050209 | *Parastrongyloides trichosuri* |  |  |  |
| X54252 | *Caenorhabditis elegans* | Rhabditoidea | V |  |
| GQ888717 | *Strongylus vulgaris* | Strongyloidea |  |  |
| NC026868 | *Strongylus equinus* |  |  |  |
| FM161883 | *Oesophagostomum quadrispinulatum* |  |  |  |
| FM161882 | *Oesophagostomum dentatum* |  |  |  |
| AJ417719 | *Necator americanus* | Ancylostomatoidea |  |  |
| AJ417718 | *Ancylostoma duodenale* |  |  |  |
| NC034289 | *Ancylostoma tubaeforme* |  |  |  |
| NC035142 | *Ancylostoma ceylaninum* |  |  |  |
| NC033886 | *Nippostrongylus brasiliensis* | Trichostrongyloidea |  |  |
| NC029736 | *Haemonchus placei* |  |  |  |
| EU346694 | *Haemonchus contortus* |  |  |  |
| NC030332 | *Angiostrongylus malaysiensis* | Metastrongyloidea |  |  |
| GQ398121 | *Angiostrongylus cantonensis* |  |  |  |
| GQ398122 | *Angiostrongylus costaricensis* |  |  |  |
| NC018602 | *Angiostrongylus vasorum* |  |  |  |
