# Supplementary figures and images for: Evaluation and utility of mitochondrial ribosomal genes for molecular systematics of parasitic nematodes
Source: Parasit Vectors. 2020 Jul 20;13:364. doi: 10.1186/s13071-020-04242-8 (PMC7372814; doi:10.1186/s13071-020-04242-8)

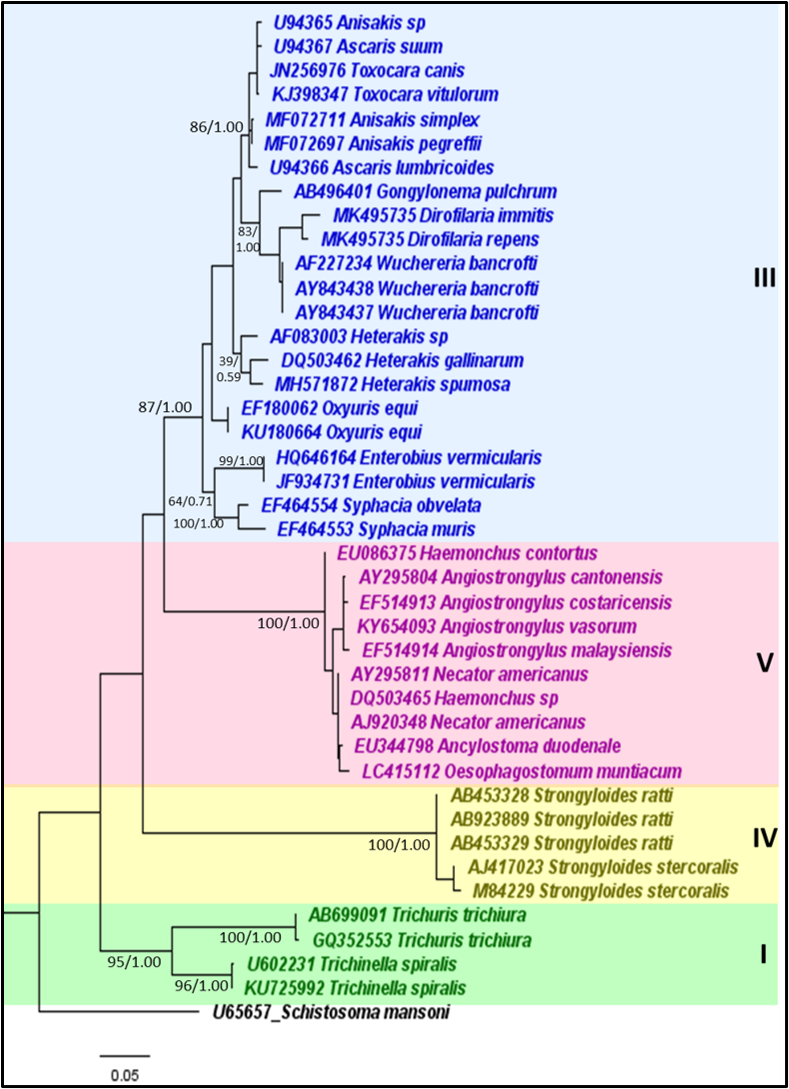

Supplement: Supplementary file 3 — Additional file 3: Figure S1. Phylogeny using 18S rRNA gene sequences as a genetic marker. Phylogenetic analyzes using maximum likelihood (TN93 + G) and Bayesian inference. Numbers at nodes indicate bootstrap values from ML analysis and posterior probability values from BI analysis (ML/BI). Different colors correspond to each nematode clade: outgroup (black); clade I (green); clade III (blue); clade IV (yellow); and clade V (purple). Monophyletic clades are highlighted in their respective color. [file 13071_2020_4242_MOESM3_ESM.tif]

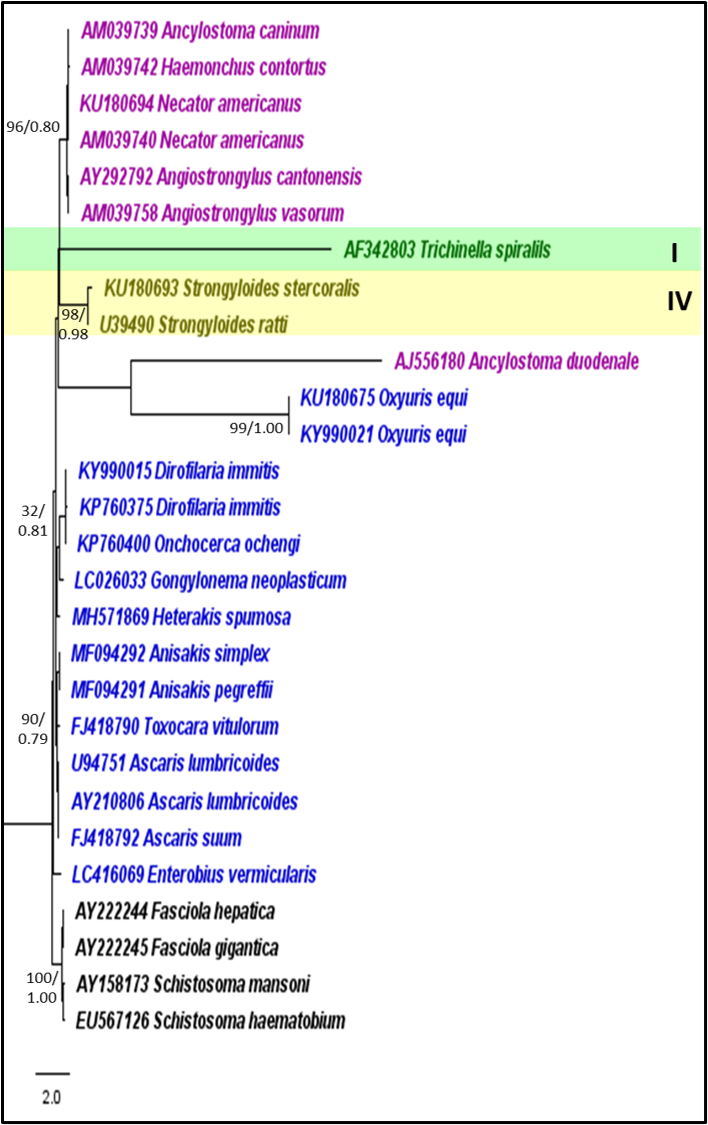

Supplement: Supplementary file 4 — Additional file 4: Figure S2. Phylogeny using 28S rRNA gene sequences as a genetic marker. Phylogenetic analyzes using maximum likelihood (TN93 + G) and Bayesian inference. Numbers at nodes indicate bootstrap values from ML analysis and posterior probability values from BI analysis (ML/BI). Different colors correspond to each nematode clade: outgroup (black); clade I (green); clade III (blue); clade IV (yellow); and clade V (purple). Monophyletic clades are highlighted in their respective color. [file 13071_2020_4242_MOESM4_ESM.tif]

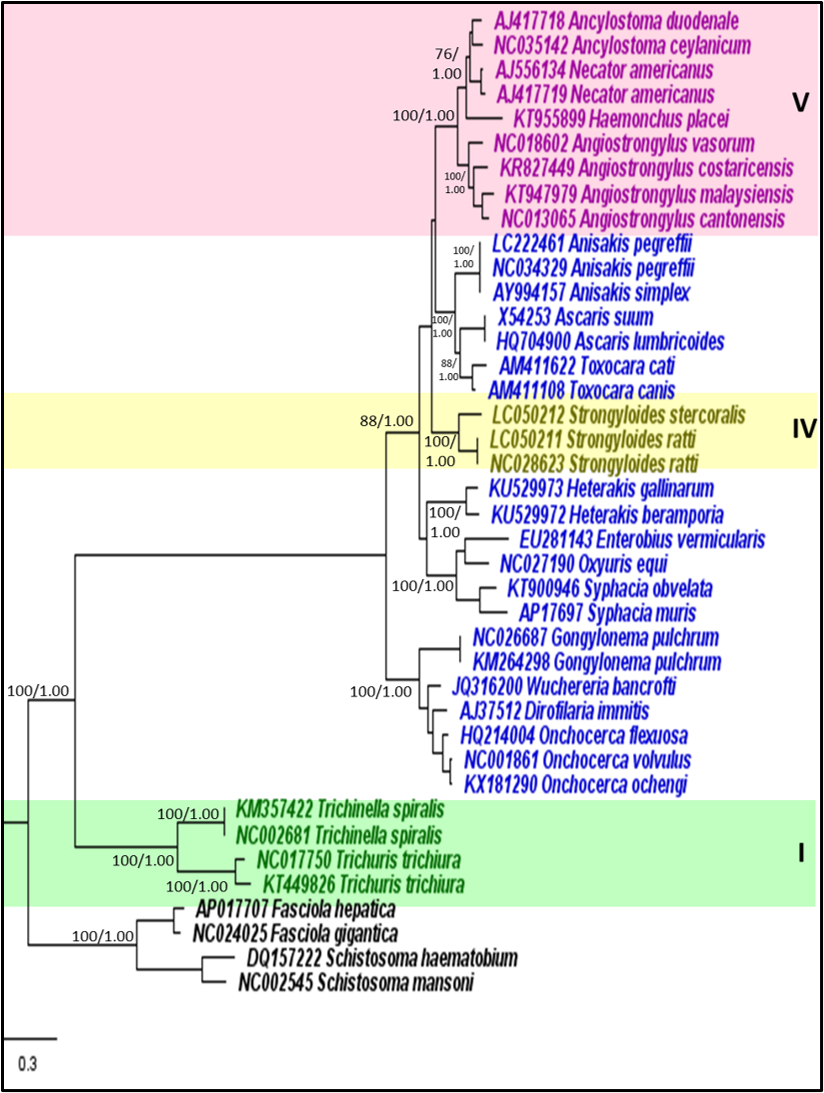

Supplement: Supplementary file 5 — Additional file 5: Figure S3. Phylogeny using cox1 gene sequences as a genetic marker. Phylogenetic analyzes using maximum likelihood (GTR + G) and Bayesian inference. Numbers at nodes indicate bootstrap values from ML analysis and posterior probability values from BI analysis (ML/BI). Different colors correspond to each nematode clade: outgroup (black); clade I (green); clade III (blue); clade IV (yellow); clade V (purple). Monophyletic clades are highlighted in their respective color. [file 13071_2020_4242_MOESM5_ESM.tif]

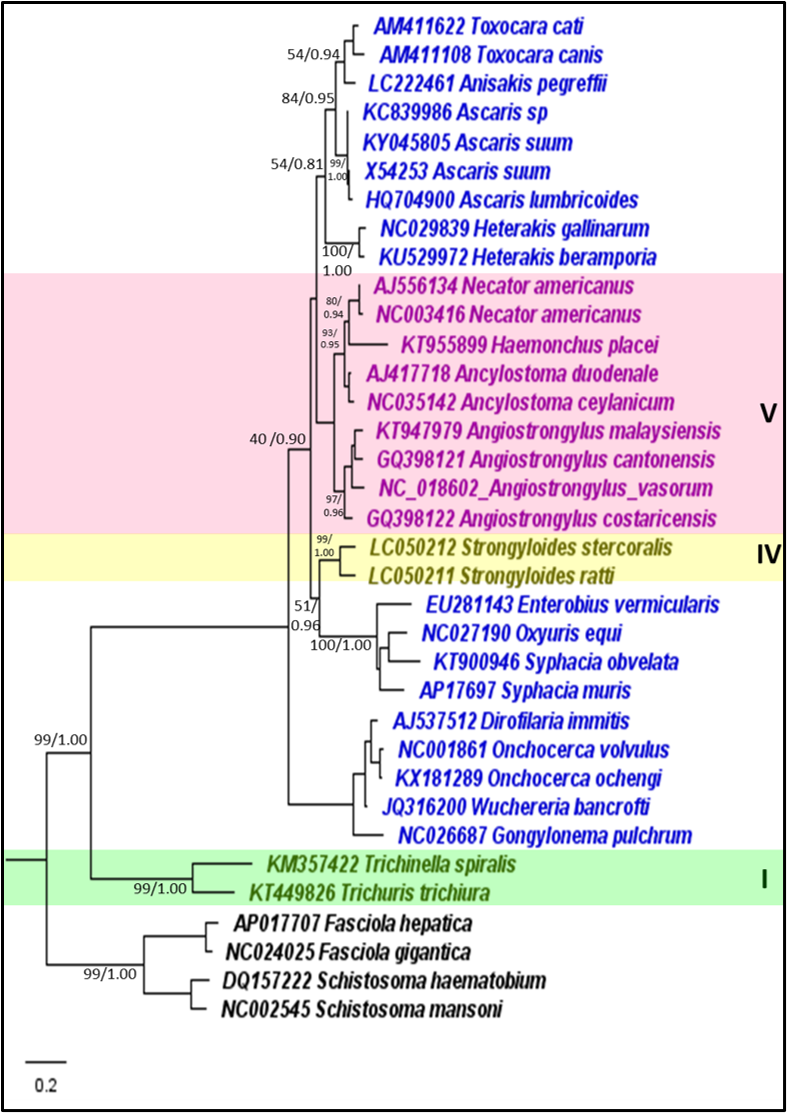

Supplement: Supplementary file 6 — Additional file 6: Figure S4. Phylogeny using 12S rRNA gene sequences as a genetic marker. Phylogenetic analyzes using maximum likelihood (HKY + G) and Bayesian inference. Numbers at nodes indicate bootstrap values from ML analysis and posterior probability values from BI analysis (ML/BI). Different colors correspond to each nematode clade: outgroup (black); clade I (green); clade III (blue); clade IV (yellow); and clade V (purple). Monophyletic clades are highlighted in their respective color. [file 13071_2020_4242_MOESM6_ESM.tif]

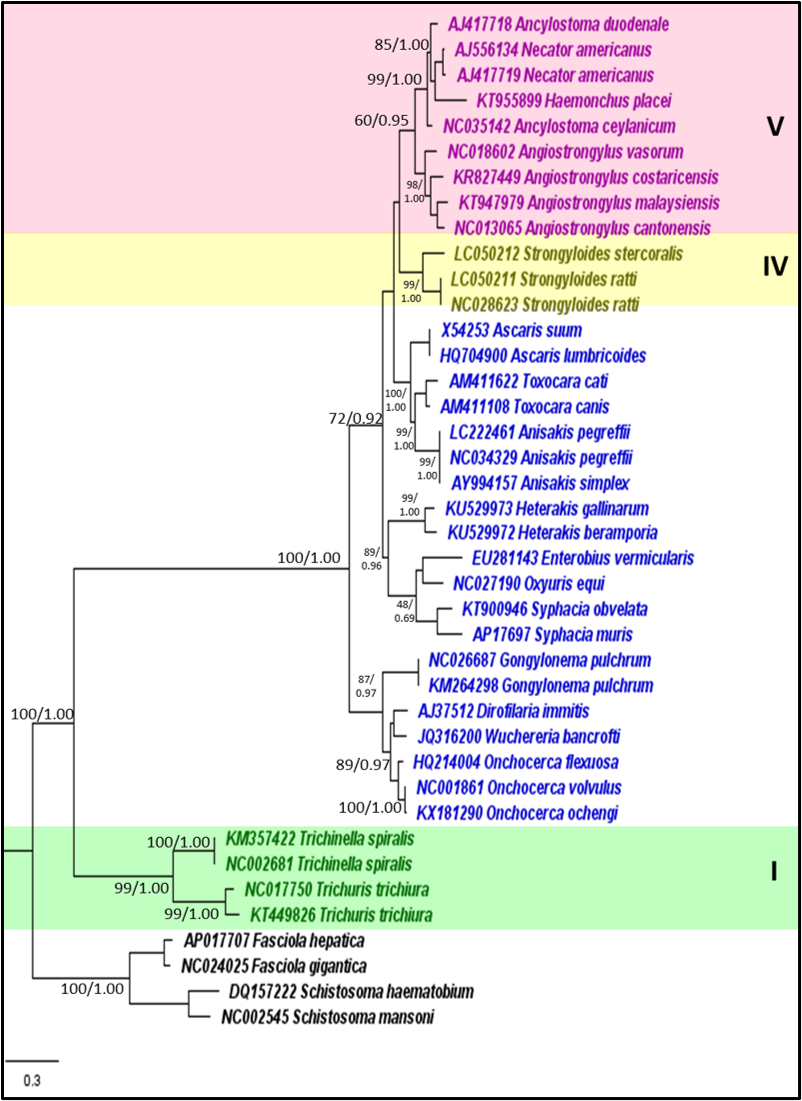

Supplement: Supplementary file 7 — Additional file 7: Figure S5. Phylogeny using 16S rRNA gene sequences as a genetic marker. Phylogenetic analyzes using maximum likelihood (GTR + G) and Bayesian inference. Numbers at nodes indicate bootstrap values from ML analysis and posterior probability values from BI analysis (ML/BI). Different colors correspond to each nematode clade: outgroup (black); clade I (green); clade III (blue); clade IV (yellow); and clade V (purple). Monophyletic clades are highlighted in their respective color. [file 13071_2020_4242_MOESM7_ESM.tif]
